# Song and speech: examining the link between singing talent and speech imitation ability

**DOI:** 10.3389/fpsyg.2013.00874

**Published:** 2013-11-22

**Authors:** Markus Christiner, Susanne M. Reiterer

**Affiliations:** ^1^Unit for Language Learning and Teaching Research, Faculty for Philological and Cultural Studies, University of ViennaVienna, Austria; ^2^English Linguistics Department, University of ViennaVienna, Austria; ^3^Centre for Teacher Education, University of ViennaVienna, Austria

**Keywords:** vocal flexibility, motor ability, singing ability, speech-sound imitation, second language pronunciation, second language acquisition, working memory, music and language

## Abstract

In previous research on speech imitation, musicality, and an ability to sing were isolated as the strongest indicators of good pronunciation skills in foreign languages. We, therefore, wanted to take a closer look at the nature of the ability to sing, which shares a common ground with the ability to imitate speech. This study focuses on whether good singing performance predicts good speech imitation. Forty-one singers of different levels of proficiency were selected for the study and their ability to sing, to imitate speech, their musical talent and working memory were tested. Results indicated that singing performance is a better indicator of the ability to imitate speech than the playing of a musical instrument. A multiple regression revealed that 64% of the speech imitation score variance could be explained by working memory together with educational background and singing performance. A second multiple regression showed that 66% of the speech imitation variance of completely unintelligible and unfamiliar language stimuli (Hindi) could be explained by working memory together with a singer's sense of rhythm and quality of voice. This supports the idea that both vocal behaviors have a common grounding in terms of vocal and motor flexibility, ontogenetic and phylogenetic development, neural orchestration and auditory memory with singing fitting better into the category of “speech” on the productive level and “music” on the acoustic level. As a result, good singers benefit from vocal and motor flexibility, productively and cognitively, in three ways. (1) Motor flexibility and the ability to sing improve language and musical function. (2) Good singers retain a certain plasticity and are open to new and unusual sound combinations during adulthood both perceptually and productively. (3) The ability to sing improves the memory span of the auditory working memory.

## Introduction

Auditory signals form the basis of human communication. The ability to correctly perceive and produce complex auditory signals is reliant upon a number of mental capacities. Typically, in foreign language acquisition, huge individual differences are observed with regards to the success rate and ultimate attainment of a learner. Increasingly, however, these individual differences in language perception and production can also be noted in the language of native speakers (Pakulak and Neville, 2010; Andringa, in press). Competent speakers of any language naturally master word stress, apply correct or functionally adequate intonation, have knowledge of sound intensity or durational patterns and use their vocal motor system effortlessly. Traditional theories surrounding the natural acquisition of the mother tongue suggest that acquisition starts immediately, even before birth (DeCasper and Fifer, 1980; McMullen and Saffran, 2004). This is the point at which, in principle, infants are open to acquiring any and “all phonetic units in language” (Kuhl, 2004). This ability is said to decrease tremendously or disappear completely after the first year of life. It is a widely held opinion that language learning becomes increasingly difficult as one ages (after critical periods) as a result of a natural tendency toward and preference to the mother tongue in terms of both perception and production. However, there are, of course, exceptions to this general “rule.” In previous research into speech imitation and pronunciation we discovered that about 15% of adult or late second language learners do not necessarily adhere to this. We labeled them speakers capable of imitating sound to a high degree (Reiterer et al., 2011). Our results showed a very clear connection between musicality in general and an ability to articulate well. The higher the musicality, the better the pronunciation and the imitation in a second language. Within the field of musicality, the ability to sing well was one of the clearest indicators of this (Nardo and Reiterer, 2009; Reiterer et al., 2011; Hu et al., 2012).

Whilst general musical training undoubtedly has an effect on an individual's creative musical outpourings, it also influences the perception and production of speech. A number of studies have already reported a positive relationship between musical competence and the processing and imitation of a foreign accent (Schön et al., 2004; Thompson et al., 2004; Wong and Perrachione, 2007; Pastuszek-Lipinska, 2008; Milovanov, 2009; Nardo and Reiterer, 2009; Kraus and Chandrasekaran, 2010; Reiterer et al., 2011; Hu et al., 2012; Christiner, 2013). Musicians, that is to say individuals with increased musical ability, show an improved auditory working memory and remember speech streams for longer when compared to those without (Pastuszek-Lipinska, 2008; Nardo and Reiterer, 2009; Reiterer et al., 2011; Hu et al., 2012; Christiner, 2013). Recent investigations have shown that the processing of verbal material and of music within the brain seem to largely overlap in the short term memory (Koelsch et al., 2009; Williamson et al., 2010; Schulze et al., 2011; Schulze and Koelsch, 2012). This may go some way to explain why musical training leads to an improvement of the short term memory when it comes to verbal material with the playing of a musical instrument and singing exercising the memory. This is not something readily practiced by non-musicians as they consume music by passive exposure alone. Publications exploring the relationship between musicality and language learning typically employ the term “musician” to refer to anyone who performs music, with an emphasis, however, on the playing of a musical instrument. As anticipated, studies focussing on the specific relationship between the ability to sing and language learning are scarce. It would seem that this musical “sub-ability” is more closely related to the ability to imitate speech rather than to competence in other musical domains. Therefore, the main focus of our investigation is this link between an ability to sing and speech imitation (pronunciation in L2 languages).

What is first important within this field, however, is to make the distinction clear between instrumentalists, on the one hand, and vocalists, on the other, as both possess markedly different musical talents. Singing should be seen as separate as, on the level of signal generation, it is based on the same principles as speech.

“Musicality” itself is a broad term that is frequently used to describe and encompass all aspects of the musical domain. As a result, the various subcategories of which the musical domain is comprised—subcategories that should be dealt with individually—are not given the proper attention. Only very recently have singing and the playing of a musical instrument been laid side by side as separate elements. This view to separate the two has, in recent years, been supported by brain imaging studies which have found that the two skill sets involved lead to increased activity in different areas of the brain (Kleber et al., 2010; Halwani et al., 2011). For example, singers showed greater complexity in certain white matter tracts resulting from their extensive vocal-motor training. This leads not only to an improvement in sound perception and production but also in feedforward and feedback control (Halwani et al., 2011). Increased activity in the primary somatosensory cortex has also been observed in the brains of classically trained singers (Kleber et al., 2010). Instrumentalists, on the other hand, showed increased activity in the primary motor cortex during performance (Lotze et al., 2003). What is most notable here is that, whilst singers improve an already established system, the vocal motor system, musicians develop additional or alternative skills (Kleber et al., 2010). Instrumentals, song and the differences between them can be assessed on two different levels: the level of acoustic perception and the level of production. Song or singing is music on the level of acoustic-perception whereas the signals produced by musical instruments differ significantly from singing in terms of generation. On the basis of signal generation, singing has a close affinity to speech. Singing teachers, for instance, argue that singing and speaking are based on the same principles such as body posture, emission, resonance and articulation, with the exception of breathing which is more active during exhalation in song than in speech (García-López and Gavilán Bouzas, 2010). A singer's enhanced vocal motor control allows them to sustain and modulate the voice effectively.

These theoretical considerations together with the results of our own preliminary investigation into the influence of singing on speech imitation (Reiterer et al., 2011; Hu et al., 2012) led to this current investigation. We hope to address the subcomponents of singing that may be involved in successful speech imitation. In previous research we had assessed the participants' ability to sing, however, we had only done this via a self-rating questionnaire. To improve on this, we tested singers of different levels of ability in further detail by means of an independent evaluation. This was for the purpose of seeing if we could replicate and/or develop the earlier results.

When testing someone's ability to sing, it is of importance to draw on the opinions and acquired knowledge of voice experts as respected professionals in their field. In most behavioral studies singing is often reduced to the generation of a melody in test conditions. These conditions do not properly examine a singer's vocal motor ability and range because the simple repetition of a familiar melody and the carrying of a basic tune is said to be manageable for most (Dalla Bella et al., 2009, 2012). Although generation of melody in this way does not effectively display a singer's full potential it may be of some use when evaluating pitch stability (Dalla Bella et al., 2007). Singing with lyrics or with certain consonant-vowel combinations, on the other hand, is a more complex task (Racette and Peretz, 2007). Singing with lyrics demonstrates a singer's vocal motor ability and their full vocal range to which the evaluation criteria of voice experts can be applied (for specific criteria see Omori et al., 1996; Ekholm et al., 1998). Singing with lyrics helps to address more of the evaluation criteria in a single singing task (Larrouy-Maestri et al., 2013). Learning and then singing a new song (both melody and lyrics) gives us insight into the recognition and memorization of song despite these areas are still not fully understood. On a very fundamental level, song consists of one or both of the following: melody and lyrics (Crowder et al., 1990). However, the question as to whether being able to memorize or recall a song involves a dual system of storage, lyrics, and melody being stored independently, remains unanswered and a topic that continues to be discussed at length (Bonnel et al., 2001; Steinke et al., 2001; Peretz et al., 2004; Racette and Peretz, 2007; Stahl et al., 2011).

We included in our investigation the singing evaluation criteria used by experts to evaluate singing from a multidimensional perspective. We had a focus on vocal motor ability (flexibility, vocal range), voice quality (resonance, warmth, and color), creativity, intonation (melody), and sense of rhythm. This helped us gauge a singer's abilities. Further to this, we carried out a variety of speech imitation tasks. We then compared this to their ability to sing and their working memory skills. The aim was to go beyond previous L2 research which had, to this point, focussed mainly on music perception and its effect on the production and memorization of language (Schön et al., 2004; Thompson et al., 2004; Wong and Perrachione, 2007; Pastuszek-Lipinska, 2008; Milovanov, 2009; Kraus and Chandrasekaran, 2010).

## Materials and methods

### Participants

In this study we selected 41 singers of different levels of ability ranging from beginners to advanced, seven of whom were male and thirty four of whom were female (aged 17–59; mean = 35.27; *SD* = 11.39). They had received formal singing lessons and, therefore, had some level of basic vocal training including knowledge of breathing exercises and breathing techniques. 75% of the participants sang regularly each week, including vocal exercises and singing lessons. 17% of them were members of a choir and 14.6% were front singers of a band. 50% of the participants had attended singing lessons for longer than three years while the remaining 50% had received singing lessons for less than this time (median). One criterion for the participation in our study was that the participants received at least three months of vocal instruction from an independent professional prior to the event. Furthermore, all participants were native German speakers who had learnt English as a second language at about the age of nine. Two of the participants were bilingual (German/English and German/Filipino), 29.3% knew only one additional or second language (English), 34.1% spoke two foreign languages (English, French, Spanish), 12.2% knew three or four languages, 4.9% spoke five languages, 4.9% spoke six languages, and 2.4% had mastered seven languages to varying degrees. None of the participants had prior experience of Hindi or, to their knowledge, been exposed to the Hindi language in any way.

### Behavioral testing 1: speech imitation

In our behavioral testing we analyzed the participants' ability to sing and imitate speech. We did this in two different ways. The first way was to test their ability to spontaneously read and repeat unknown (English) and unintelligible utterances (Hindi and non-words). Secondly, we tested their practiced abilities in both singing and speech imitation (pronunciation of a foreign language). The Hindi and non-words served as baseline stimuli resembling learning conditions without educational influence.

The speech imitation and reading tasks in English and Hindi, as well as the singing tasks, were recorded in a studio with the music software Steinberg Cubase 4. During the speech imitation tasks the participants were invited to read the well-known Aesop fable “The North Wind and the Sun” in their best English accent (British or American). They were offered some time to practice before the recording took place. In the speech imitation tasks, which did not allow practice, the participants had to repeat English and Hindi 11-syllable sentences. Hindi, as a language completely unfamiliar to all participants, tested their ability to spontaneously imitate language.

The original Hindi sentences were recorded in a sound-proof room and spoken by a native Hindi speaker. In the same way, the original English sentences were performed by American or British-English speakers. The participants began the task only after having listened to the foreign utterances three times. This was proven to be most efficient and effective following a pilot experiment. The sound files of the recordings were converted to MP3 files because the assessment was performed online. All raters rated under the same conditions. The raters were instructed to use headphones, to rate immediately after listening to a file and were able to adjust the volume on their own. The stimuli from the English imitation task and the reading of the “North Wind and the Sun” were graded by seven native English speakers and the Hindi imitation tasks by seven native speakers of Hindi. The raters were non-expert raters. However, their judgements are comparable to those of phonetic experts (Bongaerts et al., 1995; Bongaerts, 1999). The raters were instructed to judge whether the speakers sounded native-like or not (with a focus on accuracy of intonation, global speech rate, fluency, and intelligibility). The raters indicated their response on a scale of 0–10 (whereby ten was the highest and most native-like score). The first five recordings were spoken by people who were independent of the evaluation process and this functioned as a familiarization task. These had no bearing on the final result. Judges were instructed to rate files in one sitting. We ensured that each session lasted no longer than 30 min. Each of the English judges sat through two sittings because total rating time was already over 1 h. The program did not permit the skipping of a file. This ensured that all files were rated by the judge.

### Behavioral testing 2: singing skills

**Figure 1:**
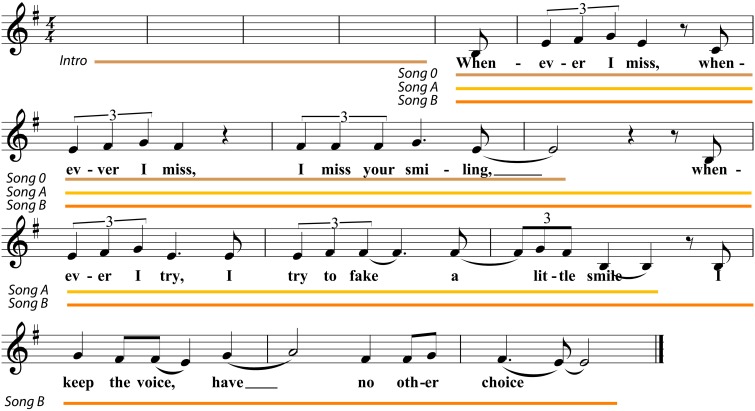
**The lyrics of the unknown song**. This figure represents the text and the song *Whenever* which was unknown to all participants. The first part (the brown line 0) was not part of the evaluation as it was a familiarization task and was performed by all participants easily. For further analysis we took the songs A (the yellow line) and B (the orange line). The latter one was remarkably longer and complex to remember.

The singing tasks consisted of different sub-tasks. When it came to learning parts of a song, the participants listened to short pieces of a newly composed song three times (lyrics in English). These pieces were unknown to them (see Figure 1). This task was divided into three conditions of increasing difficulty, which forced the participants to memories increasingly long parts of the song's lyrics, melody and rhythmic changes (see Figure 1). The first part of the newly composed song was excluded from the final analysis as it served familiarization purposes. The introductory part of the song consisted of a couple of chords (tune without lyrics) for the participants to familiarize themselves with the song's harmonies and to give them the adequate time to prepare. The participants then repeated the parts of the songs, without background music or introduction, immediately after having listened to them for a third time. The second singing task was to perform the well-known song “Happy Birthday” in a way they liked best. We did not restrict their creativity. The reason for having chosen “Happy Birthday” was that we assumed it would be familiar to the majority of our participants. Key was not part of the evaluation criteria in either singing task as the participants were instructed to sing in a key that they found pleasurable and suitable for their own singing voice. In terms of the system of evaluation, the audio files were converted into MP3 format and scored online by seven singing teachers. When rating the singing files we opted for expert raters, as, in the field of singing, unprofessional ratings are rarely seen, except for trained singing voices where it has been demonstrated that “… trained singers and non-singers did not differ significantly in their abilities to evaluate support” (Sonninen et al., 2005). We, therefore, decided on expert judgement because the tasks required expert knowledge. In the unpracticed singing tasks, the judges assessed the participants' ability to remember song lyrics, their quality of voice (warmth, color, and resonance), their sense of rhythm and how well they reproduced the original melody (pitch). The same framework for evaluation applied to the song “Happy Birthday.” Again, the highest/best score that someone could receive was 10 and the lowest 0. The raters evaluated the performances online and received login details and a password. The program did not permit the skipping of a file. This ensured that all files were rated by the judge. Judges were instructed to rate the files in a single sitting. We ensured that each session lasted no longer than 30 min. The raters received three logins—one for each task—as the overall rating process would have lasted too long. The three singing tasks received different letters: A (song A), B (song B), and C (song C). Song A had to be learnt and repeated after having listened to it for the third time. The same applies to B which was longer than song A (see Figure 1). Song C was the familiar “Happy Birthday” singing task.

### Behavioral testing 3: musicality test (AMMA) and working memory tests (WM, WM2)

In order to test the musical talent of the participants we used the AMMA test (Advanced Measures of Music Audiation, Gordon, 1989). This measured their musical abilities purely perceptually. The AMMA is a test designed for high school students and college/university music and non-music majors. The subjects have to detect either rhythmical or tonal differences in paired musical statements. The differences occur at different points and the subjects have to decide which type of differences occur, having heard the musical statements only once. In this study, all musical statements were online. Further to this, we used a working memory test (WM) (Wechsler, 1939) in order to test the subjects' working memory. The working memory test (WM) was composed of a digit span forward and a digit span backward sub-test in which the subjects had to repeat strings of numbers. In addition to this, we also tested the participants' ability to recall and repeat German non-words (WM2) (Benner, 2005).

### Behavioral testing 4: questionnaire

To the testing that we had already carried out we added a further questionnaire to *elicit* the participants' personal background, social status, and singing behavior during their childhood. The questionnaire consisted of two parts. Part one comprised questions about the participant's musical expertise, singing experience, level of musical/singing training, the musical instruments they played, L2 proficiency, educational background, L2 onset, and number of L2s spoken. In part two we introduced a multi-item scales concept (Dörnyei and Taguchi, 2010) asking participants about their singing behavior in childhood. This was carried out to assess who sang more or less often. We used more than one statement to refer to this concept. The questionnaire was performed online and the participants received login details. The computer program did not allow participants to skip questions. On average the online questionnaire lasted 25–30 min.

## Results

### Behavioral results 1

We calculated the mean of the scores for each participant and task. The mean of the Hindi imitation scores of our German speaking participants was 4.08, *SD* = 1.22. The lowest score was 1.29 and the highest was 7.14 (the scale ranging from 0 to 10). None of them were judged as being of a native-like level. This level would have been reflected in a score between 8 and 10. 2. The mean of the English imitation scores was 6.26, *SD* = 2.06. The lowest score was 1.43 and the highest was 9.14. 3. This was similar for the reading task “The North Wind and the Sun” as the mean of 7.06, *SD* = 1.17 and the scores ranged between 3.43 and 9.00. The difference between the English and the Hindi scores shows the reliability of the data as the higher means and higher maximum scores in English are an indicator that the participants had a higher proficiency in English than in Hindi. The pronunciation score referred to as speech imitation (SI) in the final analysis is the sum of the individual scores in the speech imitation tasks. The speech imitation mean is always marked with (SI). The mean of the unpracticed singing task song A was 6.08, *SD* = 1.16 and the minimum score was 3.18 and the maximum score 8.15. 3. The mean of the second unpracticed singing task song B was 5.68, *SD* = 1.15 and the scores ranged between 2.30 and 7.52. 4. The mean and minimum score of the singing task song B was considerably lower than that of singing task A. Song B was more complicated and the longest. The mean of the singing task song C “Happy Birthday” was 6.41, *SD* = 0.97 and the minimum score was 4.19, maximum score 8.08. The sum of three singing scores, quality of voice, melody, and rhythm were taken and used for further analysis. The mean of the three songs is referred to as singing ability (SA). The singing criteria creativity and remembering the lyrics were taken as separate variables. The most important variables are illustrated in Table 1 below. The dependent variables and their correlations are demonstrated in the following sections.

**Table 1:** **Illustrates the most important variables and correlations of this study**.

		**Correlations (spearman)**									
		**Speech imitation**	**Singing ability**	**Working memory (F+B)**	**Working memory non-words**	**Musicality**	**Education score**	**Sum of L2**	**English imitation**	**English text reading**	**Hindi imitation**
Speech imitation	*r*_*s*_	1	0.57^**^	0.64^**^	0.48^**^	0.32^*^	0.43^**^	0.20	0.80^**^	0.61^**^	0.87^**^
Singing ability	*r*_*s*_	0.57^**^	1	0.44^**^	0.16	0.37^**^	0.28^*^	−0.16	0.49^**^	0.23	0.46^**^
Working memory (F+B)	*r*_*s*_	0.64^**^	0.44^**^	1	0.57^**^	0.52^**^	0.17	0.23	0.47^**^	0.27^*^	0.63^**^
Working memory non-words	*r*_*s*_	0.48^**^	0.16	0.57^**^	1	0.40^**^	0.08	0.25	0.32^*^	0.37^**^	0.42^**^
Musicality	*r*_*s*_	0.32^*^	0.37^**^	0.52^**^	0.40^**^	1	0.17	0.09	0.38^**^	0.15	0.25
Education score	*r*_*s*_	0.43^**^	0.28^*^	0.17	0.08	0.17	1	0.35^*^	0.29^*^	0.34^*^	0.40^**^
Sum of L2	*r*_*s*_	0.20	−0.16	0.23	0.25	0.09	0.35^*^	1	0.06	0.25	0.19
English imitation	*r*_*s*_	0.80^**^	0.49^**^	0.47^**^	0.32^*^	0.38^**^	0.29^*^	0.06	1	0.50^**^	0.50^**^
English text reading	*r*_*s*_	0.61^**^	0.23	0.27^*^	0.37^**^	0.15	0.34^*^	0.25	0.50^**^	1	0.36^*^
Hindi imitation	*r*_*s*_	0.87^**^	0.46^**^	0.63^**^	0.42^**^	0.25	0.40^**^	0.19	0.50^**^	0.36^*^	1

** *Correlation is significant at the 0.01 level (1-tailed)*.

* *Correlation is significant at the 0.05 level (1-tailed)*.

### Speech imitation ability (SI)

The speech imitation ability (SI) was significantly correlated with the working memory test (WM), *r*_*s*_ = 0.64, *p* (one-tailed) < 0.01, and the SA, *r*_*s*_ = 0.57, *p* (one-tailed) < 0.01. There was a significant relationship between the working memory non-words repetition test (WM2), *r*_*s*_ = 0.48, *p* (one-tailed) < 0.01, the education score, *r*_*s*_ = 0.43, *p* (one-tailed) < 0.01, and the AMMA test, *r*_*s*_ = 0.32, *p* (one-tailed) < 0.05. The number of languages spoken and the L2 onset were not correlated with the speech imitation ability (SI), *p* > 0.05.

As regards the individual tasks, the English speech imitation task was significantly correlated with the Hindi speech imitation task, *r*_*s*_ = 0.50, *p* (one-tailed) < 0.01, and the English reading task, *r*_*s*_ = 0.50, *p* (one-tailed) < 0.01 as well as the working memory non-words repetition test (WM2), *r*_*s*_ = 0.32, *p* (one-tailed) < 0.05.

### Hindi

The Hindi imitation task was significantly correlated with the working memory test (WM), *r*_*s*_ = 0.63, *p* (one-tailed) < 0.01 and the singing sub-component rhythm, *r*_*s*_ = 0.53, *p* (one-tailed) < 0.01. The sub-component melody was significantly related to how well the participants repeated Hindi, *r*_*s*_ = 0.46, *p* (one-tailed) < 0.01 and how well they sang (SA), *r*_*s*_ = 0.46, *p* (one-tailed) < 0.01. Furthermore, Hindi was significantly correlated with the singing parameter quality of voice, *r*_*s*_ = 0.36, *p* (one-tailed) < 0.05.

### Singing ability (SA)

The singing ability (SA) was correlated with the speech imitation ability (SI) *r*_*s*_ = 0.57, *p* (one-tailed) < 0.01 and the English imitation task, *r*_*s*_ = 0.49, *p* (one-tailed) < 0.01. In addition, the SA was significantly related to how well the participants imitated Hindi, *r*_*s*_ = 0.46, *p* (one-tailed) < 0.01. The SA was significantly correlated with the working memory test (WM),*r*_*s*_ = 0.44, *p* (one-tailed) < 0.01. Furthermore, the SA was significantly related to the AMMA test, *r*_*s*_ = 0.37, *p* (one-tailed) < 0.01 and the psychological concept singing during childhood, *r*_*s*_ = 0.37, *p* (one-tailed) < 0.01. Singing hours per week was related to how well the participants performed in the non-words working memory task (WM2) *r*_*s*_ = 0.33, *p* (one-tailed) < 0.05.

### Singing subcomponents

Melody: The subcomponents melody of song A and B were significantly correlated with the working memory test (WM). Song A was significantly correlated with the working memory test (WM), *r*_*s*_ = 0.50, *p* (one-tailed) < 0.01_*s*_ and song B, *r*_*s*_ = 0.47, *p* (one-tailed) < 0.01.

Quality of voice: The subcomponent quality of voice was significantly correlated with the concept singing behavior during childhood, *r*_*s*_ = 0.45, *p* (one-tailed) < 0.01, and the Hindi imitation performance, *r*_*s*_ = 0.36, *p* (one-tailed) < 0.05. Text: The subcomponent text was significantly related to the working memory test (WM), *r*_*s*_ = 0.32, *p* (one-tailed) < 0.05. Creativity: The subcomponent creativity was significantly related to the singing lessons in years, *r*_*s*_ = 0.35, *p* (one-tailed) < 0.05.

### Working memory (WM)

The working memory test (WM) was significantly related to how well the participants imitated Hindi *r*_*s*_ = 0.63, *p* (one-tailed) < 0.01. Furthermore, it was significantly correlated with the English imitation task, *r*_*s*_ = 0.47, *p* (one-tailed) < 0.01. The working memory test (WM) was related to the English reading task *r*_*s*_ = 0.27, *p* (one-tailed) < 0.05.

There was a significant relationship between the musicality parameters of the AMMA test and the working memory test (WM). The working memory (WM) was correlated with the tonal discrimination ability, *r*_*s*_ = 0.45, *p* (one-tailed) < 0.01; with the rhythmic discrimination ability, *r*_*s*_ = 0.58, *p* (one-tailed) < 0.01 and with the total score of the AMMA test, *r*_*s*_ = 0.52, *p* (one-tailed) < 0.01. The working memory test (WM) was significantly related to how well the participants sang (SA), *r*_*s*_ = 0.44, *p* (one-tailed) < 0.01. The individual subcomponents of singing contribute also differently to the working memory test (WM). There was a significant relationship between the singing parameter melody, *r*_*s*_ = 0.47, *p* (one-tailed) < 0.01 and the working memory test (WM). Furthermore, the working memory test (WM) was significantly correlated with the singing parameter rhythm, *r*_*s*_ = 0.40, *p* (one-tailed) < 0.01. The working memory test (WM) was significantly correlated to the singing parameter quality of voice, *r*_*s*_ = 0.38, *p* (one-tailed) < 0.01 and was also related to how well the participants remembered the lyrics of the unknown songs A and B, *r*_*s*_ = 0.32, *p* (one-tailed) < 0.05.

### Behavioral results 2: multiple regression (MLR 1)

**Figure 2:**
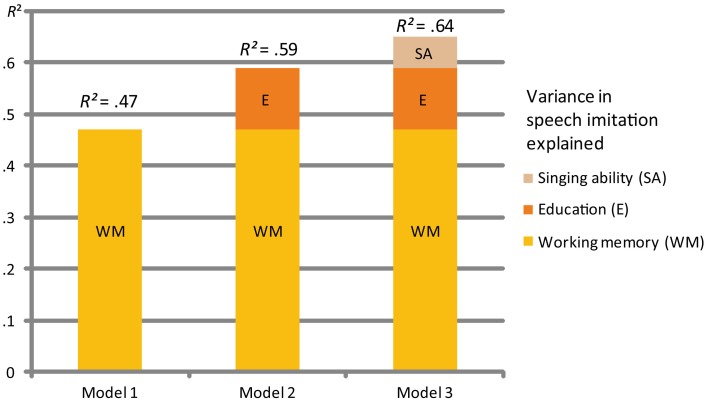
**Multiple regression MLR 1**. This figure shows the three models explaining the variance in the speech imitation ability of the participants. WM = working memory test, SA = singing ability, and E = Education score.

Having statistically isolated and characterized the relations between singing, musical expertise, and speech imitation, we wanted to know which skills were most relevant for good L2 pronunciation. All variables were entered into a stepwise multiple linear regression analysis as independent variables. The ability to imitate speech (SI) was the dependent variable. The order in which we entered the independent variables into the MLR depended on their statistical contribution in explaining the variation in the dependent variable. The criterion when entering independent variables was a probability of F-change <0.05. All variables except working memory (WM), education score (E), and singing ability (SA) were excluded as they did not contribute significantly to the probability of F-change. These three crucial factors were able to explain 64% of the variability of the speech imitation score (SI). Despite the high level of correlation with the speech imitation score (SI), the non-words working memory repetition test (WM2), the AMMA musicality test and the number of musical instruments played were not relevant for explaining the participants' ability to imitate speech (Figure 2; Table 2).

**Table 2:** **Multiple regression MLR 1**.

	**Model summary**							
	***R***	***R*^*2*^**	**F change**	**Sig. F change**	***B***	***SE B***	**β**	***p***
**Model 1**	0.68	0.47	34.45	0.000				
Constant					1.34	0.70		
Working memory (WM)					0.80	0.14	0.68	<0.01
**Model 2**	0.77	0.59	10.90	0.002				
Constant					0.24	0.71		
Working memory (WM)					0.73	0.12	0.63	<0.01
Education score (E)					0.64	0.19	0.35	<0.01
**Model 3**	0.80	0.64	5.88	0.020				
Constant					−0.92	0.82		
Working memory (WM)					0.62	0.12	0.54	<0.01
Education score (E)					0.55	0.19	0.30	<0.01
Singing ability (SA)					0.31	0.13	0.26	<0.05

*Dependent variable: Speech imitation (SI)*.

*This table shows the results of the stepwise multiple regression MLR 1. The dependent variable was the speech imitation ability (SI). The independent variables were the working memory (sometimes also called auditory short term memory) (WM), the singing ability (SA) and the education score (E)*.

### Behavioral results 3: multiple regression (MLR 2)

**Figure 3:**
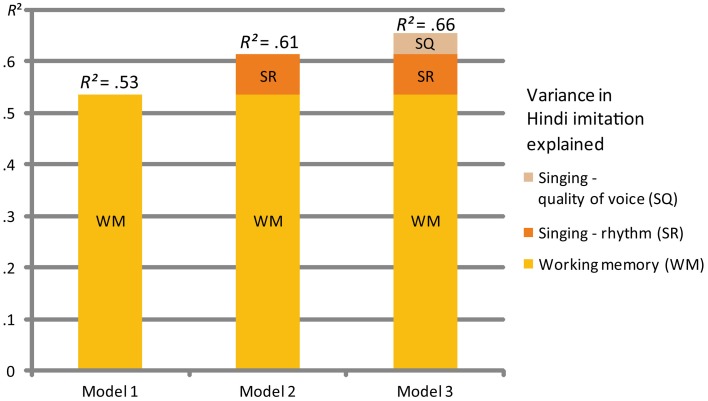
**Multiple regression MLR 2**. This figure shows the three models explaining the variance of the Hindi imitation task of the participants. WM = working memory test, SR = singing criteria: rhythm, SQ = quality of voice.

In a second stepwise multiple regression we used the Hindi score (H) as dependent variable. The order of entering the independent variables into the MLR depended on their statistical contribution when explaining the variance in the dependent variable. The criterion when entering independent variables was a probability of F-change <0.05. All variables except working memory (WM), the singing parameters rhythm (RS), and quality of voice (QS) were excluded as they did not show a significant contribution to the probability of F-change. These three factors were able to explain 66% of the variance of the imitation ability to repeat Hindi (H), the language which was previously unknown to the participants (see Figure 3; Table 3).

**Table 3:** **Multiple regression MLR 2**.

	**Model summary**							
	***R***	***R*^*2*^**	**F change**	**Sig. F change**	***B***	***SE B***	**β**	***p***
**Model 1**	0.73	0.53	44.52	0.000				
Constant					−0.46	0.69		
Working memory (WM)					0.90	0.13	0.73	<0.01
**Model 2**	0.78	0.61	7.78	0.008				
Constant					−2.76	1.04		
Working memory (WM)					0.78	0.13	0.63	<0.01
Rhythm mean of all 3 songs					0.47	0.17	0.30	<0.01
**Model 3**	0.81	0.66	4.67	0.037				
Constant					−3.43	1.04		
Working memory (WM)					0.81	0.13	0.66	<0.01
Rhythm mean of all 3 songs					0.91	0.26	0.58	<0.01
Quality mean of all 3 songs					−0.38	0.18	−0.36	<0.05

*Dependent variable: Hindi imitation*.

*This table shows the results of the stepwise multiple regression MLR 2. The dependent variable was the Hindi imitation (H). The independent variables were the working memory (WM), the singing criteria rhythm (SR), and the singing criteria quality of voice (SQ)*.

## Discussion

We found that working memory (WM), singing ability (SA), and the educational background (E) can be considered to be most central when it comes to speech imitation (SI) as demonstrated in the linear multiple regression MLR 1 (see Figure 2; Table 2). The MLR 2, however, showed different results (see Figure 3; Table 3). The education score played no role as Hindi, the dependent variable, was completely unknown to the participants. The imitation of Hindi was also the task which should have eliminated any pre-educational influence. Results indicated that working memory (WM), again, is most essential for explaining the Hindi imitation variance together with two further sub-components of singing: rhythm (SR) and quality of voice (SQ).

In the light of these results, several aspects are relevant for discussion. To better understand singing it should be approached from two viewpoints: perception and production. Additionally, we discuss the role that the working memory plays in foreign language acquisition processes and why the ability to sing leads to an improvement in the working memory.

### Perception vs. production

Generally speaking, singing shows stronger correlations to speech imitation (SI) than musicality measured purely perceptually (AMMA test). This suggests that the ability to sing is a good indicator of the ability to imitate speech. This supports previous investigations based on self-rating scales (Nardo and Reiterer, 2009; Reiterer et al., 2011; Hu et al., 2012). This finding should trigger a reconsideration of the classification of singing as one of “the nine content standards in music” (Jaffurs, 2004) as singing as behavioral practice seems more likely to be a subcategory of speech. Although musicality, on the level of acoustic-perception (AMMA test) correlated with the speech imitation performance, it was irrelevant for explaining the variance of the speech imitation performances (SI and Hindi imitation) in the multiple regressions (MLR 1 and MLR 2). The number of musical instruments the participants of this study played as well as the age at which they took up a musical instrument showed no correlation. The same was true for the musical instruments played and the participants' singing performances. Of course, it could be argued that the reason why these factors did not contribute to the participants' ability to sing and imitate speech was that the majority of the participants were not highly trained instrumentalists. This, however, further stresses that the ability to sing is a skill quite removed from the playing of a musical instrument. Singing appears more similar to music acoustically while it is closer to speech on the level of the production/generation of the signal itself.

The superiority of melody in song, on the level of acoustic-perception, becomes clear when looking at how lyrics are treated, namely, as inferior to melody. Foreign musical pieces are emotionally intelligible although listeners fail to understand the lyrics (Balkwill and Thompson, 1999). In marked contrast, if all of the phonemes of a speech act were replaced by a vowel such as /a/ while speech melody is retained, the utterance would become unintelligible (Patel, 2008). Interestingly, poetry is not categorized as song, although it shows strong rhythmic organization, structurally resembling music more than speech. This depends on the fact that language is based on timbre and music is based on pitch (Patel, 2008; Reiterer et al., 2008). In fact, several researchers favor a dual memory store for song (e.g., Bonnel et al., 2001; Peretz et al., 2004; Racette and Peretz, 2007; Stahl et al., 2011). The basic acoustic properties of music and speech (pitch and timbre) are more salient on the level of acoustic-perception. This dominance of perception over production might explain why, for instance, singing (which is “melody driven”) is more likely to be subcategorized as music. Poetry instead (which is “language driven”) falls into the category of language. Singing as a “hybrid category,” however, is musical training on the level of acoustic-perception while on the level of production it is a refined version of speech depending on enhanced vocal motor control.

### Production performance in singing and speaking

Singing and speaking are underpinned by the same speech generation process. It is very likely, therefore, that the excellent speech imitation ability of a good singer stems from their vocal flexibility which might be the result of their physical training and articulo-anatomical endowment. Halwani et al. (2011), for example, set singers aside from average people and musicians, because good singers are either aware of the sound production processes or are in the possession of special skills or talents.

A good singer displays a vocal apparatus with a good set up and a fine tuning of the palate, the tongue and the lips (Colton et al., 2006) as well as the larynx. Singing and speaking share the same sensory network and vocal apparatus. Singing and speech also share the same proprioceptive feedback system which might be more relevant to and more refined in singers. The DIVA model, for instance, proposes that speech production is controlled by “… an auditory feedback control subsystem, a somatosensory feedback control system, and a feedforward control subsystem” (Guenther, 2006). Professional singers can compensate for a lack of auditory feedback with their refined kinesthetic system and awareness for the vocal tract. This causes singers, more than non-singers and instrumentalists, to rely on the internal model during vocal production (Jones and Keough, 2008).

In general, the laryngeal motor cortex shows bihemispheric brain activation during controlled breathing conditions. This would indicate that all learnt vocal behaviors draw on common grounds whilst innate vocalizations such as laughter have a different neural control (the anterior cingulate cortex) (Simonyan et al., 2009). The neural correlates of the supralaryngeal movements include the “sensorimotor cortex […], the supplementary motor area and the superior cerebellar hemispheres” (Grabski et al., 2012b) on both hemispheres as well as orofacial motor control in the central sulcus, rostral region of the precentral gyrus, and the caudal areas in the postcentral gyrus bilaterally (Grabski et al., 2012a). Singing and speaking show bilateral activation in the inferior pre- and postcentral gyrus, the superior temporal gyrus, and the superior temporal sulcus (Özdemir et al., 2006). This would indicate that the vocalization of speech and song share largely the same neural network.

### Singing and language learning

L2 languages are not always acquired in the same way as L1 languages, especially when L2s are learnt in a formal school setting or acquired in L2 surroundings in an untutored way. In an institutionalized setting, L2 acquisition is, by and large, more concerned with the study of vocabulary and grammar than with pronunciation and the phonetic aspects of language. Consequently, language learners lack experience of how to generate L2 languages with their vocal apparatus.

L1 learners have a tendency to experiment with their vocal apparatus more than L2 learners. Firstly, the input infants receive from adults is exaggerated, simplified and highlighted and more song-like in its nature. There is a greater variation of pitch, longer vowels and/or slower pace (McMullen and Saffran, 2004) and, therefore, the language directed to infants is acoustically different to that the one directed to adults (Kuhl et al., 1997). Secondly, this language input is also linked to the motoric experience, because exaggeration or highlighting in language is not an auditory phenomenon alone but also a motoric one. This increases an infant's motor awareness and ability. This is one of the most obvious differences between L2 and L1 learning. It might be one reason why L2 acquisition is less successful than L1 acquisition. In marked contrast to L2 training, singing education is similar to L1 acquisition as it aims to create awareness about one's vocal apparatus and one's orofacial motor abilities.

Singing exercises include various combinations of non-sense intoned utterances as singers work to optimize the use of their voice. This can be seen as a general training resulting in openness to unfamiliar sounds, larger vocal range, higher vocal flexibility, and finally better speech imitation. This is reflected in our results that the non-word working memory test showed a medium correlation to the singing hours per week. Furthermore, in the MLR 2, voice quality contributed to the variance of the Hindi imitation. However, voice quality also showed a significant correlation with a participant's singing behavior during childhood, suggesting that the quality of voice is either an early developed skill or requires constant or a certain amount of time to be developed. The latter is also reported by singing professionals who propose that after four years of singing instruction singers are more proficient (Omori et al., 1996).

### Perception influenced by the production of vocalization

L1 research has shown that language acquisition develops alongside motor control, which, in turn, influences an infant's skill in expanding and developing their oral language performance (Iverson, 2010). Evidence showing that motor commands of the vocal apparatus influence language perception comes from recent proprioceptive learning tasks. In an experiment Nasir and Ostry (2009) developed a robotic device which applied a mechanical load to the jaw and displaced the natural position of the jaw whilst participants were asked to articulate certain utterances. Results demonstrated that the participants who adapted to the new motor commands showed a perceptual shift while those who did not showed no perceptual shift (Nasir and Ostry, 2009).

Similar effects have been observed in professional singers. Brain imaging studies found that long-term vocal training not only leads to “… structural adaptations in the arcuate fasciculus” (Halwani et al., 2011) and improves the interplay between the auditory feedback system and the kinesthetic system (Kleber et al., 2010), but also increases the connectivity between the somatosensory feedback system and feedback information (Halwani et al., 2011). This highlights that production influences perception in both speaking and singing. Vocal flexibility and expertise might indirectly heighten one's receptivity to new and unfamiliar sound combinations. In the present study, MLR 2 has shown that the singing parameter rhythm had a bearing on the performances in the Hindi imitation task. This would indicate that the ability to sing helps one detect rhythmic cues in foreign languages. It is likely that professional singers are more sensitive to detecting the rhythmic structures of foreign languages even if they are unintelligible—an ability which is essential for speech segmentation as well as for extracting temporal and suprasegmental information.

During the Hindi performance, participants could not rely on long-term memory retrieval as that is mainly involved in semantic coding (Baddeley, 1966, 2003). Instead they had to remember Hindi acoustically in the auditory working memory. As expected, Hindi imitation showed the strongest correlation to working memory (WM) and was *the* indicator of speech imitation ability in the MLR 1 and MLR 2. This demonstrates that the ability to repeat foreign languages is largely dependent on auditory working memory (WM).

### Working memory (WM) of singers and musicians

It is said that the auditory working memory is reliant on a phonological loop (Baddeley, 2003; Rota and Reiterer, 2009). This is described as “… [a] phonological store which can hold memory traces for a few seconds before they fade combined with an articulatory rehearsal process that makes use of subvocal speech” (Baddeley, 2003). The items remembered are limited and retrieval slows down as the number of syllables or word length increases. The capacity of the auditory working memory significantly influences language acquisition. L2 learners need to remember and repeat acoustically transported utterances they have never heard before.

Recent investigations have shown that, for instance, children who listened to music showed an improvement in their verbal ability (Moreno et al., 2011). Several studies have even reported that musicians showed a remarkably better working memory than non-musicians (Pastuszek-Lipinska, 2008; Nardo and Reiterer, 2009; Reiterer et al., 2011; Hu et al., 2012). Behavioral studies (e.g., Williamson et al., 2010) and brain imaging studies (e.g., Koelsch et al., 2009; Schulze et al., 2011; Schulze and Koelsch, 2012) found that the neural processing of tonal stimuli (including sung syllables) and verbal stimuli overlap strongly, because the working memory “… for phonemes and for pitch relies [considerably] on sensorimotor-related circuits” (Koelsch et al., 2009). Schulze and Koelsch (2012), for instance, propose that “functional plasticity is induced by music.”

The enhanced working memory of singers and musicians could also be a result of their tendency to rehearse. Usually, the longer the reproduced utterances (e.g., the Hindi imitation task comprised 11 syllables), the more likely the interruption of rehearsal by one's own auditory feedback. Singers, for instance, can sing in tune in the absence of their own auditory feedback and, at the same time, they are interrupted less by the auditory events of competing acoustic input (Sundberg, 1987). Although a familiar song, for instance, is largely recalled and stored in long term memory, the working memory is stressed for monitoring competing musical instruments. Both vocalists and musicians have to reach a compromise between attention and signal processing which, in turn, could lead to the improvement of their ability to rehearse and, ultimately, to an increased memory span.

## Conclusion

Ontogenetic and phylogenetic development, neural orchestration, auditory memory, proprioception, and sensorimotor vocal flexibility seem to be largely shared by both singing and the ability to imitate speech. In our study, the ability to sing turned out to be a good indicator of the ability to imitate speech well. Singing showed stronger correlations to speech imitation than to musicality measured perceptually. Singing, as a subcategory of music, seems to deny its close relation to speech and recent brain imaging studies would support the idea that singers should be categorized as different from instrumentalists (Kleber et al., 2010; Halwani et al., 2011). The ability to sing is a good indicator of the ability to remember new and unintelligible utterances. It can be concluded that singing training could be applied to teaching foreign and second language pronunciation as singers are in the possession of an enhanced auditory working memory and vocal flexibility. This suggests that the ability to sing speeds up that rate at which one acquires speech. Good singers retain perceptual plasticity and are open to new and unusual sound combinations throughout adulthood.

### Conflict of interest statement

The authors declare that the research was conducted in the absence of any commercial or financial relationships that could be construed as a potential conflict of interest.
